# Brand Cigarillos — A Cheap and Less Harmful Alternative to Cigarettes? Particulate Matter Emissions Suggest Otherwise

**DOI:** 10.3390/ijerph120100428

**Published:** 2015-01-06

**Authors:** Alexander Gerber, Alexander Bigelow, Michaela Schulze, David A. Groneberg

**Affiliations:** Institute of Occupational Medicine, Social Medicine and Environmental Medicine, Goethe-University, Theodor-Stern-Kai 7, Haus 9b, 60590 Frankfurt am Main, Germany; E-Mails: occup-med@uni-frankfurt.de (A.B.); Michaela.Schulze@stud.uni-frankfurt.de (M.S.); groneberg@med.uni-frankfurt.de (D.A.G.)

**Keywords:** particulate matter, tobacco, smoke, cigarette, cigarillo

## Abstract

*Background*: Environmental tobacco smoke (ETS)-associated particulate matter (PM) constitutes a considerable health risk for passive smokers. It ought to be assessed separately from the other known toxic compounds of tobacco smoke. Brand-specific differences between cigarettes and particularly between cigarettes and favorably taxed cigarillos, are of public interest and therefore worth being investigated. *Methods*: An automatic environmental tobacco smoke emitter (AETSE) was developed to generate cigarette and cigarillo smoke in a reliable and reproducible way. John Player Special (JPS) Red cigarettes, JPS filter cigarillos and 3R4F standard research cigarettes were smoked automatically in a 2.88 m^3^ glass chamber according to a standardized protocol until 5 cm from the top were burned down. *Results*: Mean concentrations (C_mean_) and area of the curve (AUC) of PM_2.5_ were measured and compared. C_mean _PM_2.5_ were found to be 804 µg/m^3 ^for 3R4F reference cigarettes, 1633 µg/m^3^ for JPS cigarettes, and 1059 µg/m^3 ^for JPS filter cigarillos. AUC PM_2.5_-values are 433,873 µg/m^3^×s for 3R4F reference cigarettes, 534,267 µg/m^3^×s for JPS Red cigarettes and 782,850 µg/m^3^×s for JPS filter cigarillos. *Conclusion*: Potential brand-specific differences of ETS-associated PM emissions among brands of cigarettes, and between cigarettes and cigarillos of the same brand and size should be investigated and published. Information about relative PM-emissions should be printed on the package.

## 1. Introduction

In recent times, cigarette manufacturers have expanded their range of products by offering cigarillos listed under the same name as their established cigarettes brands. These cigarillos are generally far less expensive than cigarettes of the same brand, as this newer kind of product is taxed lower within the European Union. In times of rising cigarette taxes, smokers who buy these cigarillos are able to stick to their usual brand instead of switching to a cheaper brand or reducing consumption, as a study describes for mainly low- income smokers [1,2]. In Europe, for example, the price of one packet JPS Red cigarettes (19 cigarettes) is 5-EUR, compared to 2.20 EUR for a packet of JPS filter cigarillos (17 cigarillos).

Environmental tobacco smoke (ETS) is a well-studied health risk not only for smokers but also for unwillingly exposed passive smokers, particularly for children [3,4]. Particulate matter (PM) has been established as a suitable parameter for measuring the environmental impact of ETS [5,6]. PM is associated with an increase of lung cancer incidences and many other respiratory diseases, possibly caused by oxidative stress and/or inflammation plus DNA damage [7]. We see environmental tobacco smoke (ETS)-associated particulate matter as a key risk factor of its own that needs to be considered independently and brand-specific. JPS cigarillos burn down ad a slower pace than JPS cigarettes. Passive cigarillo smokers are therefore exposed to ETS for an even longer time than passive cigarette smokers. Tobacco companies were forced to reduce tar and nicotine yield in their cigarettes and to print tar and nicotine content on the packets [8]; we consider particulate matter emission another potential future point of action. In a non-public environment, anti-smoking legislation cannot be enforced. Voluntary consideration for others, especially for children, is needed. Better information about the damaging effects of ETS could help plead this case.

Consumers and the public should be informed about the brand-specific different amounts of ETS-associated PM that cigarettes and cigarillos generate. For this purpose, we constructed an automatic environmental tobacco smoke emitter (AETSE), which allows us to smoke cigarettes and cigarillos in a reliable and reproducible way and to compare the ETS-associated PM amounts between cigarettes and cigarillos of internationally known brands in comparison to 3R4F reference cigarettes.

## 2. Experimental Section

### 2.1. Tobacco Products

3R4F reference cigarettes (Institute of Agriculture, University of Kentucky, USA) are manufactured for scientific purpose. Tar yield amounts to 9.5 mg, and nicotine yield to 0.73 mg. One 3R4F cigarette contains 0.78 mg of tobacco, its total length is 84 mm (filter: 27 mm).

John Player & Sons was a British tobacco and cigarette manufacturer in Nottingham. The company is now part of the Imperial Tobacco Group, the fourth largest provider on the world tobacco market. The cigarette brand John Player Special (JPS) as well as the JPS filter cigarillos are manufactured by Imperial tobacco. JPS Red cigarettes contain about 10 mg of tar, and 0.9 mg of nicotine per cigarette. One JPS Red cigarette contains 0.71 mg of tobacco. Its total length is 83 mm (filter: 20 mm). Tar and nicotine amounts of the filter cigarillos have not been published as it is not required by law. One cigarillo contains about 1.2 mg of tobacco, its total length is 83 mm (filter: 0.6 cm).

### 2.2. Automatic Environmental Tobacco Smoke Emitter (AETSE)

**Figure 1:**
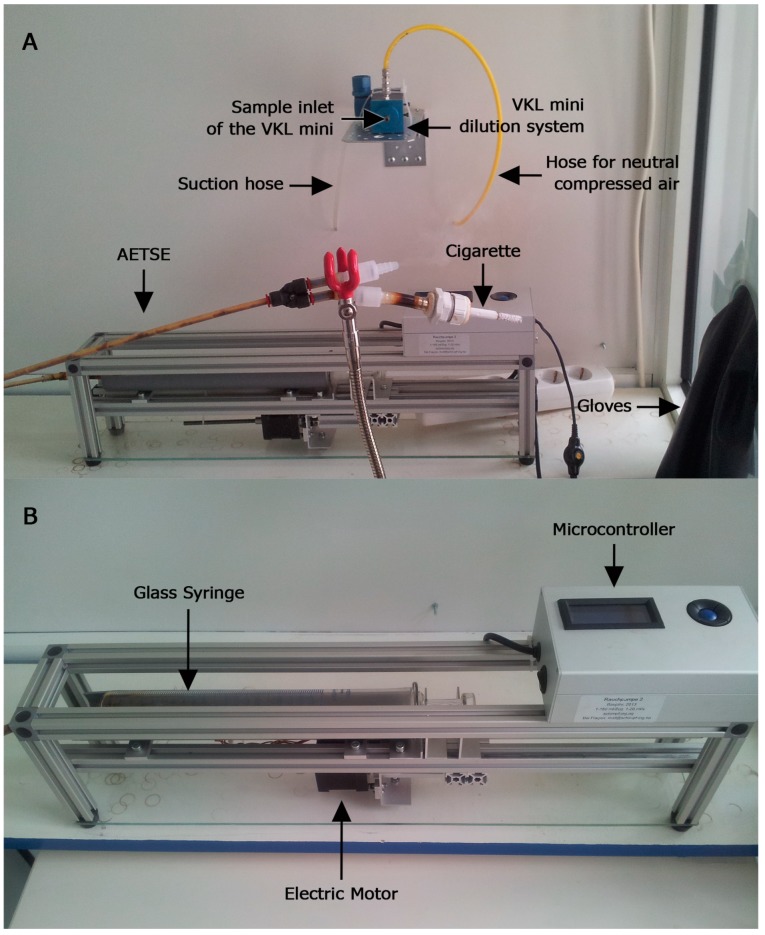
Automatic environmental tobacco smoke emitter. (**A**) Automatic environmental tobacco smoke emitter (AETSE) in the glass chamber with a smoldering cigarette. Via rubber gloves, embedded into the glass chamber, the AETSE can be operated and the cigarette can be lightened and extinguished without opening the chamber. (**B**) The AETSE consists of a glass syringe, a stepper motor, a microcontroller and stand equipment.

A predecessor model of the automatic environmental tobacco smoke emitter (AETSE) was first described in the ToPIQ study protocol [9]. This prototype had to be operated manually. Meanwhile, the AETSE was developed and constructed according to our needs by Schimpf-Ing, Trondheim (Norway), for our purpose to generate environmental tobacco smoke (ETS) in a reliable and reproducible way. It consists of a 200 mL glass syringe, a stepper motor, a microcontroller, aluminum profiles, and mechanical parts such as hoses and valves. To generate ETS, the syringe plunger is pushed and pulled by the stepper motor, thereby sucking mainstream smoke into the syringe through a non-return valve and exhaling it into the chamber through a second non-return valve, imitating puffs of a smoker (Figure 1A). Between puffs, the tobacco products continuously emit side-stream smoke. Together with the mainstream smoke, ETS is formed. To measure PM_2.5_ concentrations in a defined space, the AETSE was placed into a 2.88 m^3^ glass chamber. Two rubber gloves were fitted into one of the chamber-walls to allow access to the chamber without having to open the door and expose the researcher to harmful tobacco smoke. These gloves were used for igniting and extinguishing the tobacco products and for operating the AETSE (Figure 1B). An aerosol spectrometer (Model 11.09, Grimm Co., Ainring, Germany) was used to quantify the PM_2.5_ concentration. The aerosol spectrometer operates with a volume flow-rate of 1.2l/min. (volume controlled) and a sampling time of 6 s. To protect the measuring equipment against damage from tar and the sticky condensates of the tobacco smoke, the aerosol spectrometer was placed on a board outside the chamber, sucking the sample air from inside the chamber via a 15 cm suction hose through the back panel. Sample air was diluted pre-analytically at a ratio of 1:10, using neutral compressed air and the dilution system VKL mini (Model 7.951, Grimm Co., Ainring, Germany). The dilution system was mounted at a height of 1.70 m at the back panel of the chamber. The laboratory rooms, in which the measurements were carried out, were kept at temperatures of 22.5 °C ± 2 °C and a humidity of 29% ± 5%. Daily diverging environmental PM concentrations did not influence our measurements as these were performed inside the glass chamber in our laboratory rooms. The Glass chamber was cleaned daily, and the continuously documented baseline for PM_2.5_ was stated at 1 to 3 µg/m^3^.

### 2.3. Smoking Protocol

According to the smoking protocol we developed for this study, as many identical puffs as necessary were performed to burn off 5 cm off the length of the analyzed tobacco product. Puff duration was 3 s, puff volume 40 mL. To support ignition, the protocol started with a double-puff, followed by a 27 s smoldering phase of the tobacco product until the next puff was launched. The complete smoking cycle consisted of four phases: in the pre-ignition phase, the baseline was measured for 5 min; the combustion phase started with the ignition of the tobacco product and ended by manual extinction when 5 cm of the tobacco product had been burned; a 5 min post-combustion phase was recorded, followed by a 5 min suction phase. The tobacco products were weighed before and after combustion to calculate the mass of tobacco burnt (Table 1).

**Table 1:** Average PM_2.5_ mean concentrations, average AUC, average peak concentrations, an average combustion time and average mass of tobacco burnt for 3R4F reference cigarettes, JPS Red cigarettes and cigarillos. The Sample air was diluted pre-analytically at 1:10.

Tobacco Product	C_mean_ PM_2.5 _(µg/m^3^)	C_peak_PM_2.5_ (µg/m^3^)	AUC PM_2.5_ (µg/m^3 ^× s)	Combustion Time (s)	Average Mass of Tobacco Burnt (mg)
3R4F Reference	804 ± 79	1392 ± 150	433,873 ± 51,168	539 ± 44	702 ± 24
JPS Red Cigarettes	1633 ± 163	3113 ± 289	534,267 ± 61,992	328 ± 32	529 ± 31
JPS Cigarillos	1059 ± 389	2386 ± 856	782,850 ± 146,538	813 ± 242	741 ± 62

### 2.4. Data Processing and Analysis

**Figure 2:**
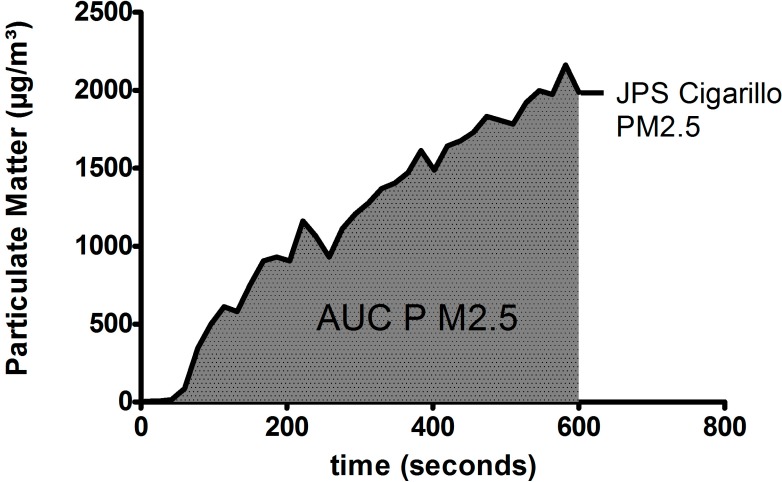
Particulate matter (PM) concentration and area under the curve (AUC) from ignition to extinction of John Player Special (JPS) cigarillos. Cigarillos were manually extinct when 5 cm from the top had burnt.

We calculated PM_2.5_ mean concentrations (C_mean_) and area under the curve (AUC) during the combustion phase only, using “Graph Pad Prism 5.03” (Figure 2). In toxico-pharmacological studies, AUC is known as the mathematical integral in a plot of drugs in blood plasma against time. We used the AUC respectively in a plot of particulate matter concentration in breathing air against time. To our knowledge, this mathematical method has not been used in literature so far in connection with ETS. We think it describes the ETS-associated PM burden vividly and we have used it already to describe the short-term but high-impact exposure of spectators during an urban building demolition [10].

**Figure 3:**
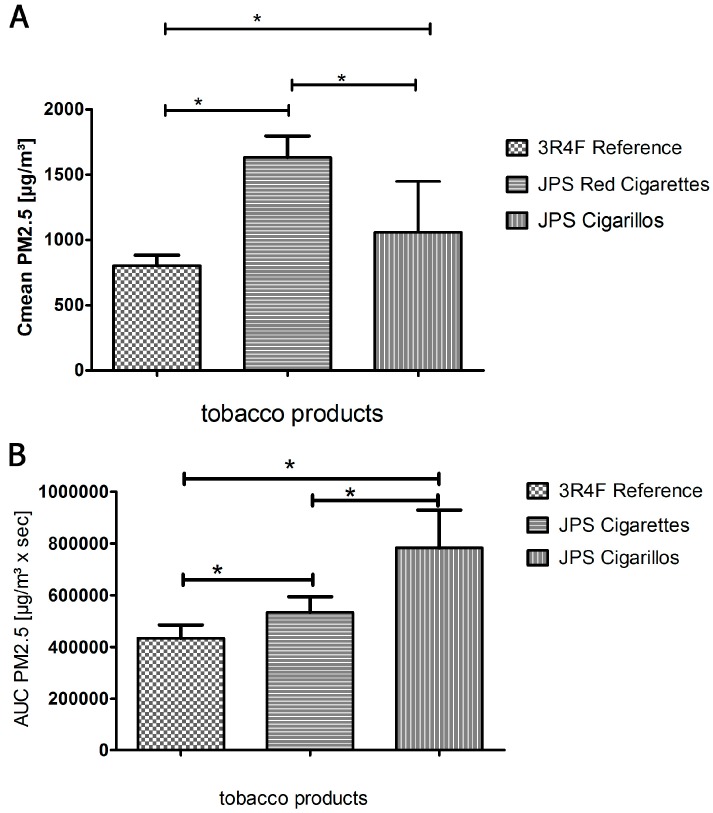
PM_2.5_ Concentrations (**A**) and AUC (**B**) for each tobacco product tested. * indicates *p* < 0.05.

**Figure 4:**
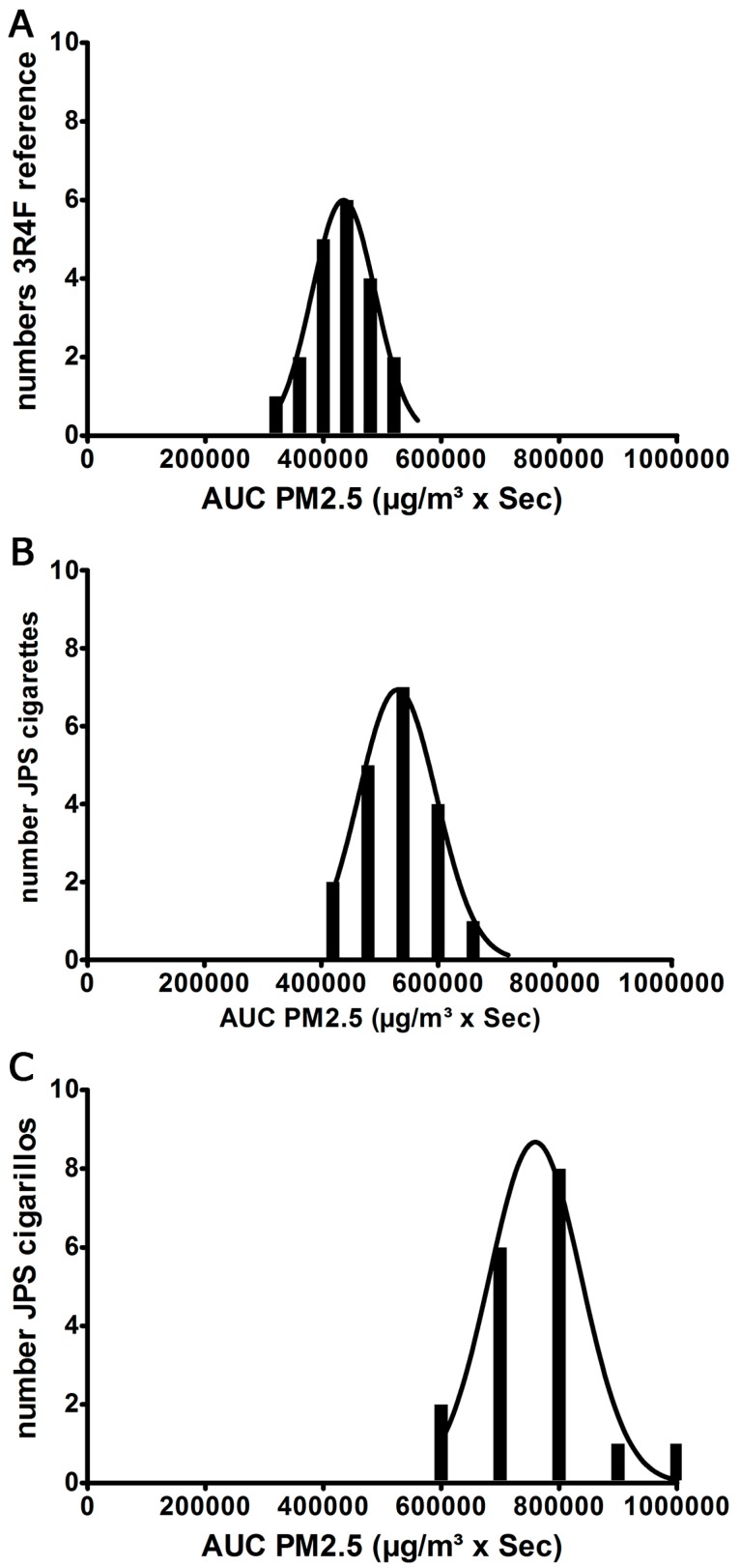
Bar chart of AUC against number of cigarettes with Gaussian curve for 3R4F reference—cigarettes (**A**), JPS cigarettes (**B**) and JPS cigarillos (**C**).

A package of 3R4F reference cigarettes (n = 20), JPS Red cigarettes, (n = 19) and JPS filter cigarillos (n = 17) were used for the tests. Significant differences between these three different types of tobacco products with respect to the PM_2.5_ emission parameters C_mean_ and AUC were assumed when the one-sample *t*-test gave a value of *p* < 0.05 (Figure 3A,B). Before performing the *t*-test, the exposure parameters C_mean_ and AUC had to be tested for a Gaussian distribution and proved to be normally distributed. Figure 4 shows the Gaussian distribution for the AUC-parameters of all tested brands (Figure 4A–C).

## 3. Results and Discussion

### 3.1. Results

We found statistically significant differences in PM_2.5_ C_mean_ and AUC (Table 1, Figure 3A,B).

Table 1 shows average PM_2.5_ mean concentrations, average AUC, average peak concentrations, an average combustion time and average mass of tobacco burnt for 3R4F reference cigarettes, JPS Red cigarettes and cigarillos. The Sample air was diluted pre-analytically at 1:10.

PM mean concentrations of JPS red cigarettes exceeded those of 3R4F reference cigarettes by more than 100%, and those of JPS filter cigarillos by more than 50%. When analyzing PM_2.5_ concentration against time of exposure (AUC), however, AUC PM_2.5_ measured data was by far highest in JPS filter cigarillos, followed by JPS cigarettes.

### 3.2. Discussion

ETS-associated PM amounts differ considerably between cigarette brands, as already shown in the ToPIQ study protocol, in particular when looking at the AUC.

Brand-specific examination of ETS and particulate pollution matters when considering that brand-loyal smokers expose their fellow humans with ETS of the same brand for decades in some cases. Particulate matter containing macrophage-activating particles and lipopolysaccharides [11,12] contributes to epithelial inflammation. This has proved to be a central component in the pathology of smoke-related lung diseases [13,14,15].

In this study we demonstrate a significant increase of PM_2.5_ emitted by JPS cigarillos compared to reference cigarettes or brand cigarettes. JPS red cigarettes emit a higher mean concentration of PM_2.5_ in a shorter amount of time. Although PM_2.5_ mean concentration is highest with the JPS Red cigarette, JPS filter cigarillos generate by far the largest amount of PM_2.5_, based on the area under the curve, which also takes into account the extended period of smoking. This effect is also seen with 3R4F reference cigarettes, generating the lowest C_mean _PM_2.5_ but a higher AUC than JPS Red cigarettes. 3R4F cigarettes took an average of 539 s to burn down 5 cm from the top, while JPS Red cigarettes took just 328 s, when smoked in the same standardized way. The difference may be due to production details, such as density of the tobacco filling, addition of substances to improve taste and burning qualities, or the content of cellulose which is used as a binding substance for the machine-made short filler tobacco. Besides these facts, the mass of tobacco burned is largest in the cigarillos (Table 1). A standard deviation (SD) of <15% respectively regarding C_mean_ and AUC PM_2.5_ was documented in reference cigarettes and JPS Red cigarettes and reflects the reliability of our method. However, we assume that a SD of 19% and 37% found in C_mean_ and AUC PM_2.5_ of JPS Cigarillos is due to the cheaper production process and shows, how difficult it is to assess cigarillo smoke.

Other groups have investigated PM_2.5_ concentrations in public spaces such as restaurants, pubs and internet cafés in Malaysia [16], or hospitals, government buildings, restaurant and entertainment venues in Seoul where smoke-free policies are quite loose [17]. Kungskulniti* et al.* assessed second-hand smoke in international airports in Thailand and compared their findings with exposure findings in international airports in the USA [6]. These groups report about PM_2.5_ concentrations between 105 and >500 µg/m^3^ in the presence of active smokers. Our findings are not in the same range. It should be noted though, that with using an AETSE and a 2.88 m^3^ glass chamber, we did not want to imitate real-life conditions or real-life smoking behavior. Smokers vary their smoking behavior individually and inter-individually, depending on time and local situation. Our results cannot represent these factual circumstances. They were not intended to provide absolute PM data for defined situations, but rather to enable a comparison of different brands and tobacco products [18,19]. For that reason we developed our own standardized smoking protocol according to our requirements. Developing the study protocol, we followed the ISO intense regime [20] in puff frequency but decided to use a smaller puff volume for technical reasons concerning our AETSE.

In a very recent publication, Vardavas* et al.* examined non-smoking employees in semi-open air cafés in Athens, Greece, and correlated their post-work shift tobacco specific 4-(methylnitrosamino)-1-(3-pyridyl)-1-butanol (NNAL) concentrations in urine samples with work shift PM_2.5_ concentrations attributable to second-hand smoke [21]. The group demonstrated that NNAL concentrations increases by 9.5%, per 10 µg/m^3^ increase in PM_2.5_ concentrations.

In the context with these findings, it should be mentioned that, in addition to inflammatory and immunologic effects, respirable and alveolar particles may also serve as transporters for carcinogenic substances with low volatility like polycyclic aromatic hydrocarbons or aromatic amines permitting their transport into distal lung areas.

Measures to reduce tar yield and nicotine are already legally required in most countries. But amongst carcinogenic and specific toxic substances contained in ETS, PM emissions also constitute an important independent risk factor and therefore, efforts should be undertaken to reduce PM.

It should be emphasized that cigarillos are not a less harmful alternative to cigarettes. Even in those smokers who do not inhale the mainstream smoke, cigarillos may have pathogenic effects on the upper respiratory track causing throat or tongue cancer. Through ETS, its associated PM and toxic compounds, cigarillos are also a health risk to involuntary second-hand smokers. These points considered, tax advantages for cigarillos compared to cigarettes are unfounded and should be abolished. Also PM amounts in relation to reference cigarettes should be printed on packages because consumers have the right to be informed about the harm they are causing to their environment.

## 4. Conclusions

Environmental tobacco smoke constitutes a major contributor to indoor air pollution in industrialized countries and causes illness and death of countless humans worldwide. In addition to specific toxic and carcinogenic compounds, particulate matter represents an independent health hazard and its amounts vary in a brand-specific way. This paper illuminates the various impact, different tobacco products and tobacco brands may have on the particulate matter exposure of passive smokers. Taking the extended burning time of cigarillos into consideration, the AUC-method is a suitable way to evaluate PM-exposure complementary to mean concentration.
